# RFtest: A Robust and Flexible Community-Level Test for Microbiome Data Powerfully Detects Phylogenetically Clustered Signals

**DOI:** 10.3389/fgene.2021.749573

**Published:** 2022-01-24

**Authors:** Lujun Zhang, Yanshan Wang, Jingwen Chen, Jun Chen

**Affiliations:** ^1^ Department of Biostatistics and Bioinformatics, Duke University School of Medicine, Durham, NC, United States; ^2^ Institute of Soil and Water Resources and Environmental Science, College of Environmental and Resource Sciences, Zhejiang University, Hangzhou, China; ^3^ Department of Health Information Management, University of Pittsburgh, Pittsburgh, PA, United States; ^4^ Department of General Surgery, Zhongshan Hospital, Fudan University, Shanghai, China; ^5^ Department of Quantitative Health Sciences, Mayo Clinic, Rochester, MN, United States

**Keywords:** random forest, hypothesis testing, community-wide test, microbiome, omics association test

## Abstract

Random forest is considered as one of the most successful machine learning algorithms, which has been widely used to construct microbiome-based predictive models. However, its use as a statistical testing method has not been explored. In this study, we propose “Random Forest Test” (RFtest), a global (community-level) test based on random forest for high-dimensional and phylogenetically structured microbiome data. RFtest is a permutation test using the generalization error of random forest as the test statistic. Our simulations demonstrate that RFtest has controlled type I error rates, that its power is superior to competing methods for phylogenetically clustered signals, and that it is robust to outliers and adaptive to interaction effects and non-linear associations. Finally, we apply RFtest to two real microbiome datasets to ascertain whether microbial communities are associated or not with the outcome variables.

## 1 Introduction

The microbiome, the collection of microorganisms and their genetic materials in an environment, has been intricately related to human health (Gao et al., 2018; Gentile and Weir, 2018) and ecosystem functioning (Fierer, 2017). Studying the composition and function of the microbiome has been greatly facilitated by next-generation sequencing *via* marker gene (Weisburg et al., 1991) and/or shotgun metagenomic sequencing techniques (Handelsman, 2004). For the past three decades, the marker gene sequencing has been the dominant approach to investigate the phylogenies and the abundance of microbial groups (Weisburg et al., 1991), while shotgun metagenomics has become increasingly popular to study the functional potential of the microbiome (Quince et al., 2017). Sequences stemming from this marker gene sequencing procedure are usually quality-filtered, merged, and clustered into operational taxonomic units (OTUs) (Schloss et al., 2009; Edgar, 2013) or denoised into amplicon sequence variants (ASVs) (Callahan et al., 2016; Bharti and Grimm, 2021). These OTUs and ASVs are regarded as surrogates of microbial taxa, and downstream statistical analyses are then performed based on the OTU/ASV abundance table, which records the frequencies of the detected OTUs/ASVs in each microbiome sample, together with a phylogenetic tree relating the OTUs/ASVs and the metadata describing the characteristics of the samples.

One central task of microbiome data analyses is to test the association between the microbiome and a variable of interest, while adjusting for potential confounders. Although the ultimate goal is to identify specific microbial taxa associated with the variable of interest, a process also known as differential abundance analysis (Chen et al., 2018), the large abundance variation, weak effects, and the need for multiple testing correction makes differential abundance analysis underpowered for a moderate sample size. It is not uncommon that differential abundance analysis fails to make any discoveries after multiple testing correction when a number of microbial taxa are weakly associated with the variable of interest. In such cases, a community-level test, which jointly analyzes the abundance data at the community level, may be more powerful due to its ability to pool individual weak signals and no need for multiple testing correction. It is also possible to explore the interspecific interactions (Zengler and Zaramela, 2018) and phylogenetic relations (Washburne et al., 2018) in the test to further improve the statistical power. In fact, the community-level tests have been routinely applied, as the first step in statistical analysis of microbiome data, to establish an overall association between the microbiome and the variable of interest. They have been instrumental in disentangling microbial association with, for example, clinical outcomes (Clooney et al., 2021) and environmental gradients (Zhang et al., 2021).

The first community-level test for microbiome data is based on permutational multivariate analysis of variance (PERMANOVA) (Anderson, 2001). PERMANOVA is a distance-based permutation test for assessing the association between a multivariate outcome and a covariate of interest, where the variability of the multivariate outcome is summarized in a distance/dissimilarity matrix. In microbiome applications, ecologically motivated distances/dissimilarities, such as UniFrac (Lozupone and Knight, 2005; Lozupone et al., 2007) distance and Bray–Curtis dissimilarity (Bray and Curtis, 1957), are frequently used. As an alternative to PERMANOVA, the microbiome regression-based kernel association test (MiRKAT) (Zhao et al., 2015) follows a similar logic but treats the abundance data as the covariate and transforms those distance or dissimilarity matrices into kernels; subsequently, community-level associations are evaluated using semi-parametric kernel machine regressions. MiRKAT is computationally efficient, allows a straightforward adjustment for covariates, and accommodates multiple distance kernels through an omnibus test (Zhao et al., 2015). Another community-level test is the adaptive microbiome-based sum of powered scores (aMiSPU), which is an adaptive test based on a series of microbiome-based sum of powered scores (MiSPU) calculated using different powers (Wu et al., 2016). aMiSPU utilizes the variable selection/weighting of the SPU framework (Pan et al., 2014) based on weighted and unweighted generalized taxon proportions and is designed to adapt to the underlying signal structure. Combining the strength of MiRKAT and aMiSPU, the optimal microbiome-based association test (OMiAT) (Koh et al., 2017) substitutes MiSPU with its non-phylogenetic version, sum of powered scores (SPU), and integrates these two criteria *via* an omnibus *p*-value to improve power. These methods all use permutation to assess the statistical significance and hence the type I error rates are well controlled (Anderson, 2001; Zhao et al., 2015; Wu et al., 2016; Koh et al., 2017). However, their power relies on the choice of candidate distances/kernels or specific data transformation (e.g., the power function for MiSPU). Moreover, they have limited ability to exploit the interactions among taxa, which are expected to be prevalent in microbiome data (Zengler and Zaramela, 2018). Additionally, they have not leveraged the strength of machine learning algorithms, which have been shown to be effective in building up microbiome-based predictive models (Marcos-Zambrano et al., 2021).

In the present study, we propose a community-level test based on random forest (RFtest) for testing the associations between the microbiome and an outcome variable. Random forest (Breiman, 2001) is considered as one of the most successful machine learning algorithms, which can be readily applied to diverse tasks, such as variable selection and prediction from high-dimensional omics datasets (Degenhardt et al., 2019). As a non-parametric decision tree-based method, it is robust to outliers and can automatically adapt to the complex relationship between the taxa abundance and the outcome variable without the need for data transformation. Moreover, they can capture high-order interactions in the data without prior knowledge provided (Wright et al., 2016). The proposed method RFtest uses the generalization error estimate of random forest as the test statistic and uses permutation to calculate *p*-values. It incorporates the phylogenetic information *via* creating features that accumulate OTU/ASV abundance along the branches of the phylogenetic tree. RFtest is flexible and can be applied to different types of outcomes. It can also adjust covariates, which facilitates confounder adjustment in microbiome association analysis. By comprehensive simulations, we show that our approach has controlled type I error rates, and is particularly powerful to detect phylogenetically clustered signal, robust to outliers, and capable of detecting complex relationships between microbial taxa, and between the taxa and the outcome.

## 2 Methods and Materials

### 2.1 Notations

Suppose that we have abundance measurements from *n* independent microbiome samples and *p* OTUs/ASVs, denoted by **X** = (**X**_1_, **X**_2_, … , **X**_*i*_, … , **X**_*n*_)^T^ (1 ≤ *i* ≤ *n*), where **X**_*i*_ = (*x*_*i*1_, *x*_*i*2_, … , *x*_*ij*_, … , *x*_*ip*_)^T^ (1 ≤ *j* ≤ *p*) and *x*_*ij*_ is the (normalized) abundance of the *j* ^th^ OTU/ASV in the *i* ^th^ sample. Let **Y** = (*y*_1_, *y*_2_, … , *y*_*i*_, … , *y*_*n*_)^T^ (1 ≤ *i* ≤ *n*) denote the vector for the outcome variable, such as clinical outcomes and environmental gradients. Additionally, we may have *q* covariates, such as age and biological sex, which are denoted by **Z**_*n*×*q*_ = (**Z**_1_, **Z**_2_, … , **Z**_*i*_, … , **Z**_*n*_)^T^, where **Z**_*i*_ = (*z*_*i*1_, *z*_*i*2_, … , *z*_*ik*_, … , *z*_*iq*_)^T^ (1 ≤ *k* ≤ *q*) are the measurement of the *q* covariates in the *i*th sample. Moreover, we may have a rooted phylogenetic tree *G* capturing the phylogenetic relatedness of the OTUs/ASVs. *G* has *p* leaves (terminal vertices with a degree of 1) and one node (an internal vertex with a degree greater than 1) called root. The *p* leaves correspond to the *p* OTUs/ASVs while the root is theoretically assumed to be the last common ancestor of all vertices in the phylogenetic tree. In a path connecting a leaf and the root, the vertices closer to the root are regarded as “ancestors” of vertices that are farther from; thus, this ancestral relationship describes the relative closeness of vertices to the root of *G*. The aim for RFtest is to test the association between **Y**_*n*×1_ and **X**_*n*×*p*_ while adjusting **Z**_*n*×*q*_.

### 2.2 Methods

The tree of life underpins our understanding towards microorganisms (Washburne et al., 2018). Closely related microorganisms share similar biological traits and association signals tend to be clustered with respect to their phylogenetic relationship (Xiao et al., 2017; Xiao et al., 2018a; Xiao et al., 2018b). We therefore aim to utilize the phylogenetic information in the random forest test to improve its power. We incorporate such phylogenetic information by augmenting the OTU/ASV-level abundance data with the abundances of the internal nodes of the phylogenetic tree *G*. This is achieved by creating an *n*-by-*m* feature matrix **W**_*n*×*m*_ = (*w*_*il*_)_*n*×*m*_ for the *m* internal nodes in *G*, where the features accumulate the abundance of OTUs/ASVs belonging to the same ancestor in *G*. As each leaf corresponds to one OTU/ASV in microbiome and there exists exactly one path between each leaf and the root, the total abundance of all OTU/ASV leaves that shares a specific common ancestor or internal node *l* is well-defined. Thus, we have

$$w_{il}=\underset{j\in A}{\sum }x_{ij}$$ (1)

where *w*_*il*_ is the collective abundance of the *l* ^th^ internal node of the *i* ^th^ sample and A is the set of OTUs/ASVs whose ancestor is the *l* ^th^ internal node.

The RFtest uses the generalization error rate estimate (Breiman, 2001) of random forest as a test statistic, and uses permutation to calculate *p*-values. Specifically, random forest is firstly grown using the “ranger” package (Wright and Ziegler, 2017) in the R platform (Team, 2020) using **Y**_*n*×1_ as outcome variable and **X**_*n*×*p*_ and **W**_*n*×*m*_ as input features, and the observed out-of-bag (OOB) error rate *T*_obs_ is used as the test statistic. The OOB error is the average error for each observation calculated using predictions from the trees that do not contain in their respective bootstrap sample. Here, we use the probabilistic prediction for classification and the OOB error is essentially a Brier’s score (Malley et al., 2012). Regression and classification trees are used for continuous and binary **Y**s, respectively. When there are no covariates, it permutes the outcome **Y**_*n*×1_ *B* times and calculates the OOB error rate ${\tilde{T}}^{b}\;\left(b=1,\;\ldots ,\;B\right)$ based on the permuted **Y**_*n*×1_. The *p*-value is calculated using:

$$p‐\text{value}=\left[\#\left({\tilde{T}}^{b}\leq T_{obs}\right)+1\right]/\left(B+1\right)$$ (2)

where #( ${\tilde{T}}^{b}$ ≤ *T*_obs_) is the number of permuted datasets satisfying ${\tilde{T}}^{b}$ ≤ *T*_obs_.

When covariates are present, RFtest accommodates covariates using the following steps. Firstly, **Y**_*n*×1_ is regressed on covariate **Z**_*k*_ (1 ≤ *k* ≤ *q*) using linear model if **Y** is continuous:

$$y_{i}={\hat{\beta }}_{0}+{\hat{\beta }}_{1}z_{i1}+\ldots +{\hat{\beta }}_{q}z_{iq}+e_{i}={\hat{\beta }}_{0}+\overset{q}{\underset{k=1}{\sum }}{\hat{\beta }}_{k}z_{ik}+e_{i}$$ (3)

and using logistic regression model if **Y** is binary:

$$\text{logit}\left(P\left(y_{i}=1\right)\right)={\hat{\beta }}_{0}+\overset{q}{\underset{k=1}{\sum }}{\hat{\beta }}_{k}z_{ik}$$ (4)

where ${\hat{\beta }}_{0}$ and ${\hat{\beta }}_{k}$ (1 ≤ *k* ≤ *q*) are the estimated coefficients, and *e*_*i*_ are regression residuals. Next, for a continuous **Y**, we generate ${\tilde{Y}}^{b}$ using residual permutation. The observed error rate *T*_obs_ is calculated based on the input features **X**_*n*×*p*_ and **W**_*n*×m_ and the adjusted outcome **Y**_adj_ = (*e*_1_, *e*_2_, … , *e*_*i*_, … , *e*_*n*_)^T^ (1 ≤ *i* ≤ *n*). Thereafter, the permutated ${\tilde{Y}}^{b}=\;{\left({\tilde{y}}_{1}^{b},{\tilde{y}}_{2}^{b},\;\ldots ,\;{\tilde{y}}_{i}^{b},\;\ldots ,\;{\tilde{y}}_{n}^{b}\right)}^{\text{T}}$ is generated by

$${\tilde{y}}_{i}^{b}={\tilde{e}}_{i}^{b}$$ (5)

where ${\tilde{e}}_{i}^{b}$ is the permutated regression residuals for the *i*th sample. For a binary covariate **Y**, ${\tilde{Y}}^{b}$ is generated using a (0, 1) random number generator according to adjusted probabilities of

$$\text{logit}\left(P\left({\tilde{y}}_{i}^{b}=1\;|\;\underset{i}{\sum }{\tilde{y}}_{i}^{b}=\;\underset{i}{\sum }y_{i}\right)\right)={\hat{\beta }}_{0}+\overset{q}{\underset{k=1}{\sum }}{\hat{\beta }}_{k}z_{ik}$$ (6)

where we conditioned on the number of observed cases. Finally, we calculate the error rate ${\tilde{T}}^{b}$ under permutation based on ${\tilde{Y}}^{b}$ similarly. Consequently, *p*-value can be obtained using (Eq. 2).

We implemented the random forest test in the package “RFtest” on the R platform, which is available on GitHub (https://github.com/Lujun995/Random-forest-test-RFtest).

### 2.3 Simulation Studies

Simulations were conducted under various scenarios to study whether RFtest would control type I error rates at desired levels and whether it would be a powerful testing approach compared with competing methods. Instead of using a parametrical model such as the Dirichlet-multinomial model (Chen and Li, 2013), the microbiome data were directly resampled from a large gut microbiome study by Hale et al. (2017). Briefly, the study compared the fecal microbiome profiles of patients with adenomas versus healthy controls. 16s rRNA sequences were analyzed using IM-TORNADO pipeline (Jeraldo et al., 2014), OTUs were clustered at 97% identity, and singletons were removed (Hale et al., 2017). After rarefaction to 20,000 counts per sample, the adenoma dataset contained 439 samples and 2,100 OTUs, where we resampled *n* = 50 samples, i.e., **X**_50×*p*_, without replacement for each simulated dataset. We then constructed the outcome variable **Y**_50×1_ under six scenarios, following the strategy by Zhao et al. (2015). Let S denote the set that comprises OTUs associated with **Y**. We generated the continuous and binary outcome **Y** = (*y*_1_, *y*_2_, … , *y*_*i*_, … , *y*_50_)^T^ (1 ≤ *i* ≤ 50) based on

$$y_{i}=\beta_{0}+z_{i}+\beta \;\;scale\left[\sum_{j\in S}\left(x_{ij}\right)\right]+\varepsilon_{i},$$ (7)

and

$$\log\mathrm{it}\left(P\left(y_{i}=1\right)\right)=\beta_{0}+z_{i}+\beta \;\;scale\left[\sum_{j\in S}\left(x_{ij}\right)\right],$$ (8)

where *β*_0_ is a constant, *β* is an adjustable effect size, *ε*_*i*_ ∼ *N* (0, *σ* ^2^), and the “scale” function standardizes the data to have mean 0 and standard deviation 1. We used *β*_0_ = 10 for a continuous **Y** and *β*_0_ = 0 for a binary **Y**, *ε*_*i*_ ∼ *N* (0, 1).

The first scenario (S0) was used to study the type I error rate of RFtest by setting the effect size *β* = 0 under three cases, including no covariates [*z*_*i*_ = 0 and *ε*_*i*_ ∼ *N* (0, 1)], one covariate independent of **X** (*z*_*i*_ ∼ *N* (0, 1) and *ε*_*i*_ ∼ *N* (0, 9)), and one covariate associated with **X** {*z*_*i*_ = scale[ $\Sigma_{j\in S}\left(x_{ij}\right)$ ] + *N* (0, 1) and *ε*_*i*_ ∼ *N* (0, 9)}, respectively. In the third case, S consisted of OTUs from an abundant lineage S _*A*_, which contributed to 15% of the total OTU number and 21% of the total abundance.

The other five scenarios (S1–S5) were used to evaluate the power of RFtest. No covariates were included (*z*_*i*_ = 0) in these scenarios. In S1, we investigated different signal types (phylogenetically clustered vs. non-phylogenetically clustered) and different signal densities (5% vs. 15%). For phylogenetically clustered signals, the signal OTUs for 5% and 15% densities were from two abundant lineages S _*B*_ and S _*A*_, respectively, where S _*B*_ was contained in S _*A*_ described above and contributed to 5% of the total OTU number and 11% of the total abundance. For non-clustered signals, the signal OTUs were randomly selected and OTUs for 5% density were also contained in those for 15%. We further substituted the term $\Sigma_{j\in S}\left(x_{ij}\right)$ in Eq. 7 and Eq. 8 with $\Sigma_{j\in S}\left(x_{ij}/\bar{x_{\cdot j}}\right)$ , where $\bar{x_{\cdot j}}=\frac{1}{n}\overset{n}{\underset{i=1}{\sum }}\left(x_{ij}\right)$ , to avoid several OTUs dominating the overall signal strength.

The scenario S2 was designed to further validate the results of clustered signals in S1 using different lineages. We studied seven disjoint major lineages ( S _*I*_: *I* = *A*, *C*, *D*, *E*, *F*, *G*, *H*) in the dataset of Hale et al. (2017), which spanned 80% of the total OTU number and more than 80% of the total abundance. Each lineage possessed 5%–20% of the total OTU number and 1%–40% of the overall abundance. The simulations in this scenario were conducted under *β* = 2.25 for a binary **Y** and *β* = 0.75 for a continuous **Y**.

The scenario S3 was to evaluate the power of the RFtest when the outcome variable was non-linearly associated with the signal OTUs. We applied a non-linear link function *f*_link_ to *x*_*ij*_. Specifically,

$$y_{i}=\beta_{0}+\beta \;\;scale\left[\sum_{j\in S}f_{link}\left(x_{ij}\right)\right]+\varepsilon_{i}$$ (9)

for a continuous **Y**, and

$$\log\mathrm{it}\left(P\left(y_{i}=1\right)\right)=\beta_{0}+\beta \;\;scale\left[\sum_{j\in S}f_{link}\left(x_{ij}\right)\right]$$ (10)

for a binary **Y**, where *f*_link_(*x*_*ij*_) = log_2_(*x*_*ij*_ + 1) (*x*_*ij*_ ≥ 0).

The scenario S4 studied a complex association between **Y** and **X** where there was interaction between two sets of signal OTUs. Particularly, for a continuous **Y**, it was generated *via*

$$y_{i}=\beta_{0}+\beta \;\;scale\left[\sum_{j\in S}\left(x_{ij}\right)\right]\cdot scale\left[\sum_{j'\in S'}\left(x_{ij'}\right)\right]+\varepsilon_{i}$$ (11)

and for a binary **Y**, it was generated using

$$\log\mathrm{it}\left(P\left(y_{i}=1\right)\right)=\beta_{0}+\beta \;scale\left[\sum_{j\in S}\left(x_{ij}\right)\right]\cdot scale\left[\sum_{j'\in S'}\left(x_{ij'}\right)\right],$$ (12)

where *β* was fixed at 1.33 and 5 for a continuous and binary **Y**, respectively, and S and S' were two disjoint sets comprising 15% and 13% of total OTUs, respectively. For phylogenetic signals, we let S = S _*A*_ and S ’ = S _*C*_, where S _*A*_ had been characterized in S0 and S _*C*_ was another major lineage accounting for 12% of the total abundance. For non-phylogenetic signal, the terms $\sum_{j\in S}$ (*x*_*ij*_) and $\sum_{j'\in S'\;}$ (*x*_*ij*’_) in Eq. 11 and Eq. 12 were normalized using $\underset{j\in S\;\text{or}\;S}{\sum }\left(x_{ij}/\bar{x_{\cdot j}}\right)$ , where $\bar{x_{\cdot j}}=\frac{1}{n}\overset{n}{\underset{i=1}{\sum }}\left(x_{ij}\right)$ . The sample size used in this scenario ranged from 50 to 250 as detection of an interaction generally requires a relatively large sample size.

The last scenario (S5) was used to assess the robustness of RFtest to outliers. Firstly, the outcome variable **Y** was generated according to the procedure in S1, using clustered or non-clustered signals with a density of 15%. Subsequently, the order of OTUs in 0, 1, or 3 samples was randomly shuffled, yielding 0, 1, or 3 outliers; therefore, these outliers would possess distinct microbiome profiles.

The source code of this section is available at GitHub (https://github.com/Lujun995/RFtest-Simulations).

### 2.4 Competing Methods and Evaluation

The competing methods include the optimal microbiome regression-based kernel association test (optimal MiRKAT) (version 1.1.1, https://cran.r-project.org/package=MiRKAT) (Zhao et al., 2015), the adaptive microbiome-based sum of powered score test (aMiSPU) (version 1.0, https://cran.r-project.org/package=MiSPU) (Wu et al., 2016) and optimal microbiome-based association test (OMiAT) (version 6.0, https://github.com/hk1785/OMiAT) (Koh et al., 2017). While multiple distance or dissimilarity functions could be used in MiRKAT, we followed the example in the “MiRKAT” package (Zhao et al., 2015) and selected weighted and unweighted UniFrac distance (Lozupone and Knight, 2005; Lozupone et al., 2007) and Bray–Curtis dissimilarities (Bray and Curtis, 1957), which have been widely used in microbiome studies. All the results were averaged over 1,000 simulation runs.

## 3 Results

### 3.1 Simulation Studies

#### 3.1.1 Factors Influencing the Power of RFtest

We first studied factors that might influence the performance of RFtest including choice of the test statistic, method for *p*-value calculation, sparsity filtering, and the parameters of the random forest (“ranger”). Results of these evaluations were obtained under the scenario S1 (binary outcome).

For the choices of test statistic, we investigated the OOB error rate (“OOB_P”), training error, 0.632 error, and 0.632 + error based on probabilistic predictions. It is well known that the training error underestimates the generalization error while OOB error overestimates it. The 0.632 and 0.632 + rule proposed by Efron and Tibshirani (Efron and Tibshirani, 1997) tried to obtain a more unbiased estimate. In addition to the use of probabilistic predictions, we also compared to the OOB error rate based on binary prediction (“OOB_noP”). Supplementary Figure S1 shows that error rates based on probability predictions were found to be more powerful than that based on binary predictions, while for different types of error rates based on probabilistic predictions, their performance was similar (Supplementary Figure S1). Thus, we selected the OOB error rate with probabilistic predictions as the test statistic. Next, we compared the permutation test to a naïve test, which applied a Wilcoxon rank sum test based on the OOB predicted probabilities. We observed that their *p*-values were highly correlated (Supplementary Figure S2); nonetheless, the naïve approach was unable to adjust for covariates and slightly less powerful than the permutation-based RFtest (Supplementary Figure S3). We also examined the effect of sparsity filtering on power and computational time of RFtest by filtering features at sparsity thresholds of 98%, 96%, 90%, and 80%. Supplementary Figure S4 shows that mild filtering (e.g., filter OTUs present in less than 4%–10% of samples) was more beneficial than no filtering or aggressive filtering. Such mild filtering could remarkably reduce computation time while maintaining a similar power. Finally, we studied the impact of the parameters of random forest (“ranger”) on the power of RFtest. Concerning the number of split variables, splitting a proportion of 2%–3% of the total OTU number (close to the default) generally performed well under both phylogenetic and non-phylogenetic signals while a greater or smaller number might be preferrable for phylogenetic or non-phylogenetic signals, respectively (Supplementary Figure S5). A larger number of decision trees in random forest would stabilize the error rate (Supplementary Figure S6A); however, the variance of the sampling distribution of the error rate under permutation was observed 10 times larger than the variance of the OOB error rate across different runs (Supplementary Figure S6A). Thus, a larger number would hardly increase the power of the RFtest (Supplementary Figure S6B) but significantly increase computational burden. Based on these evaluations, we used an ensemble of 500 decision trees in the RFtest to accelerate the computation and stabilized the estimated error rate by averaging over three runs.

#### 3.1.2 Type I Error Control

We studied the type I error rate control of RFtest by simulating null datasets (S0) with or without covariates. At the nominal level of 5%, we observed that the type I error was controlled at the desired level in situations where a covariate was absent, independent with **X** or correlated with **X** (Table 1).

**TABLE 1:** Estimated type I error rate of the random forest test (RFtest).

	Binary outcome variable (Y)	Continuous Y
No covariates (Z)	4.7% (3.6%, 6.2%)^a^	5.3% (4.1%, 6.9%)
Z independent with microbiome data (X)	5.2% (4.0%, 6.8%)	4.7% (3.6%, 6.2%)
Z correlated with X	3.6% (2.6%, 4.9%)	2.9% (2.0%, 4.1%)

a Data are presented as “proportion (*L*, *U*),” where the proportion is a point estimate of type I error rate and the *L* and the *U* are the lower and upper bounds of Wilson’s 95% confidence interval for proportion data. Type I error rates are expected to be ≤ ∼5%.

#### 3.1.3 Power Studies

**FIGURE 1:**
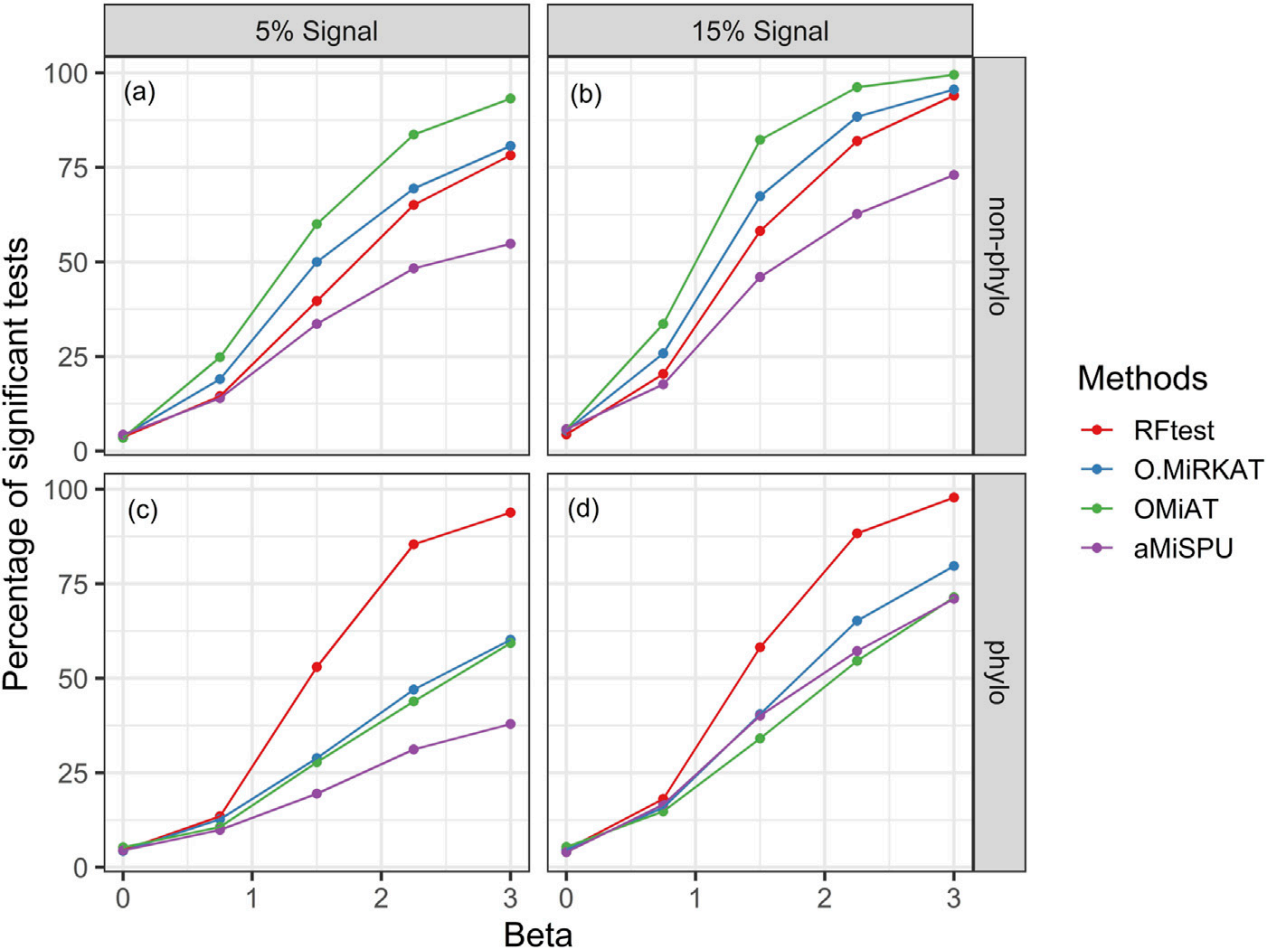
Power comparison among the competing methods for a binary outcome variable under different signal types and densities. Abbreviation: O.MiRKAT, optimal MiRKAT. **(A,B)** Random signals with a density of 5% and 15%, respectively. **(C,D)** Phylogenetically clustered signal with a density of 5% and 15%, respectively.

**FIGURE 2:**
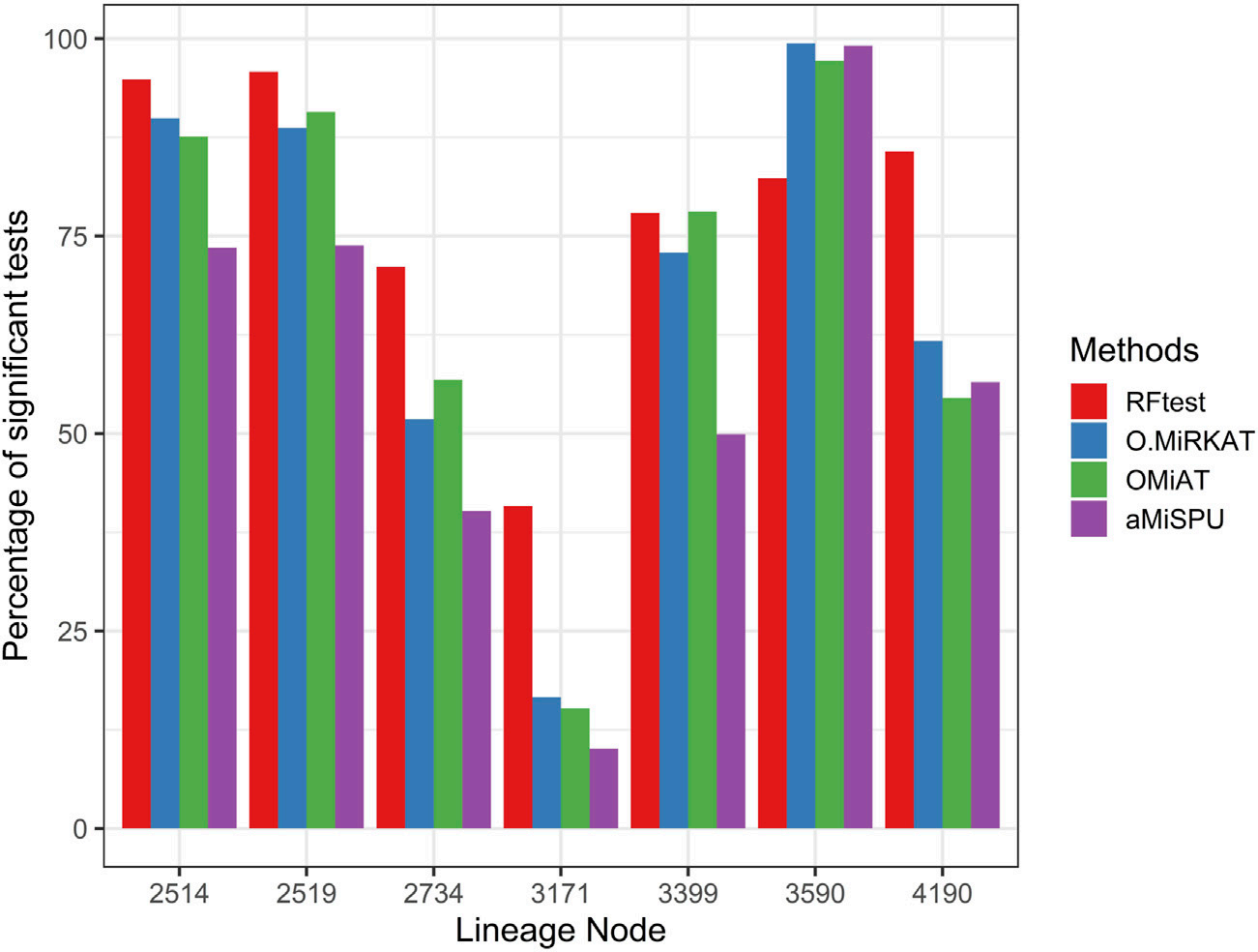
Power comparison among the four competing methods under signals from seven major lineages. The lineage numbers correspond to node numbers in the phylogenetic tree used in simulation in the present study. These lineages span ≥80% of the total OTUs and the total abundance. Abbreviations have the same meaning as in Figure 1.

Next, we studied the power of RFtest under different scenarios with association signals (S1–S5). In scenario S1, RFtest was more powerful than competing methods under phylogenetically clustered signals across signal densities for both binary and continuous outcomes (Figures 1C,D **S7c** & **S7d**). While the margin by which the RFtest led might expand or contract for different OTU clusters defined based on the phylogenetic tree in scenario S2 (Figure 2 & **S8**), RFtest was generally considered as a leading test among all competing methods except in lineage “3590” (Figure 2 & **S8**). Furthermore, this margin was more notable when the outcome variable is binary (Figures 1C,D, **S7c** & **S7d**). For random or non-phylogenetic signals, however, the RFtest appeared to be less powerful than OMiAT and optimal MiRKAT but outperformed aMiSPU (Figures 1A,B, **1b**, **S7a** & **S7b**).

**FIGURE 3:**
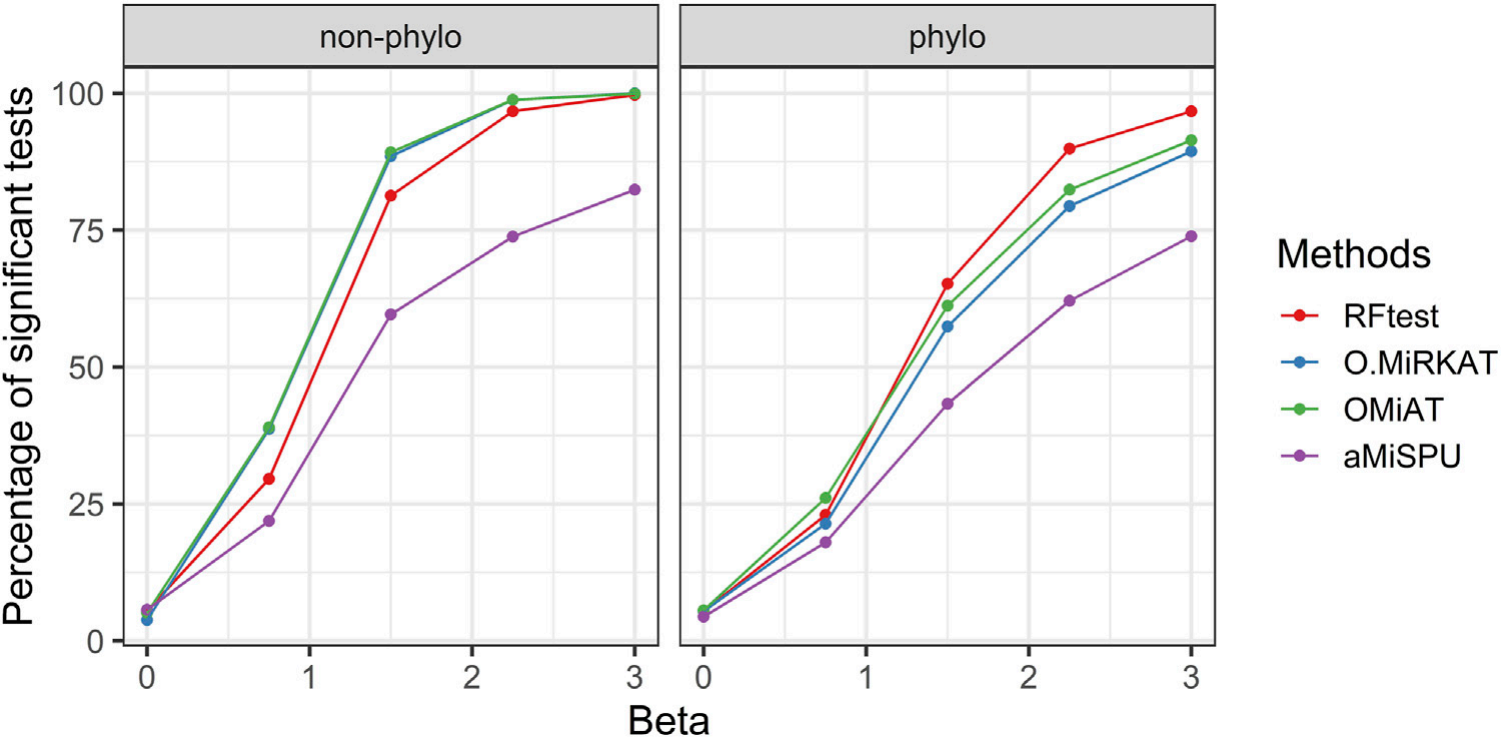
Power comparison among the competing methods for a binary outcome variable when **X** and **Y** are non-linearly associated. The raw OTU abundance data were transformed using a link function of *f*_log2_ (*x*_*ij*_) = log_2_ (*x*_*ij*_ + 1) (*x*_*ij*_ ≥ 0). Two signal types, phylogenetic and non-phylogenetic, with a density of 15% were used.

Scenarios S3–6 demonstrated the robustness of the RFtest to outliers and its adaptivity to diverse association patterns between **X** and **Y**. In scenario S3, the microbiome profile **X** was related to **Y** on the log scale yielding a non-linear relationship. We found that the results remained similar to those in scenario S1, where a linear relationship was assumed. The RFtest was observed to maintain a leading position under phylogenetical signals but became relatively less powerful under non-phylogenetic signals (Figure 3 & **S9**). However, compared to scenario S1, the difference diminished among the RFtest, the optimal MiRKAT, and the OMiAT (Figure 3 & **S9**). These three methods also outperformed aMiSPU (Figure 3 & **S9**).

**FIGURE 4:**
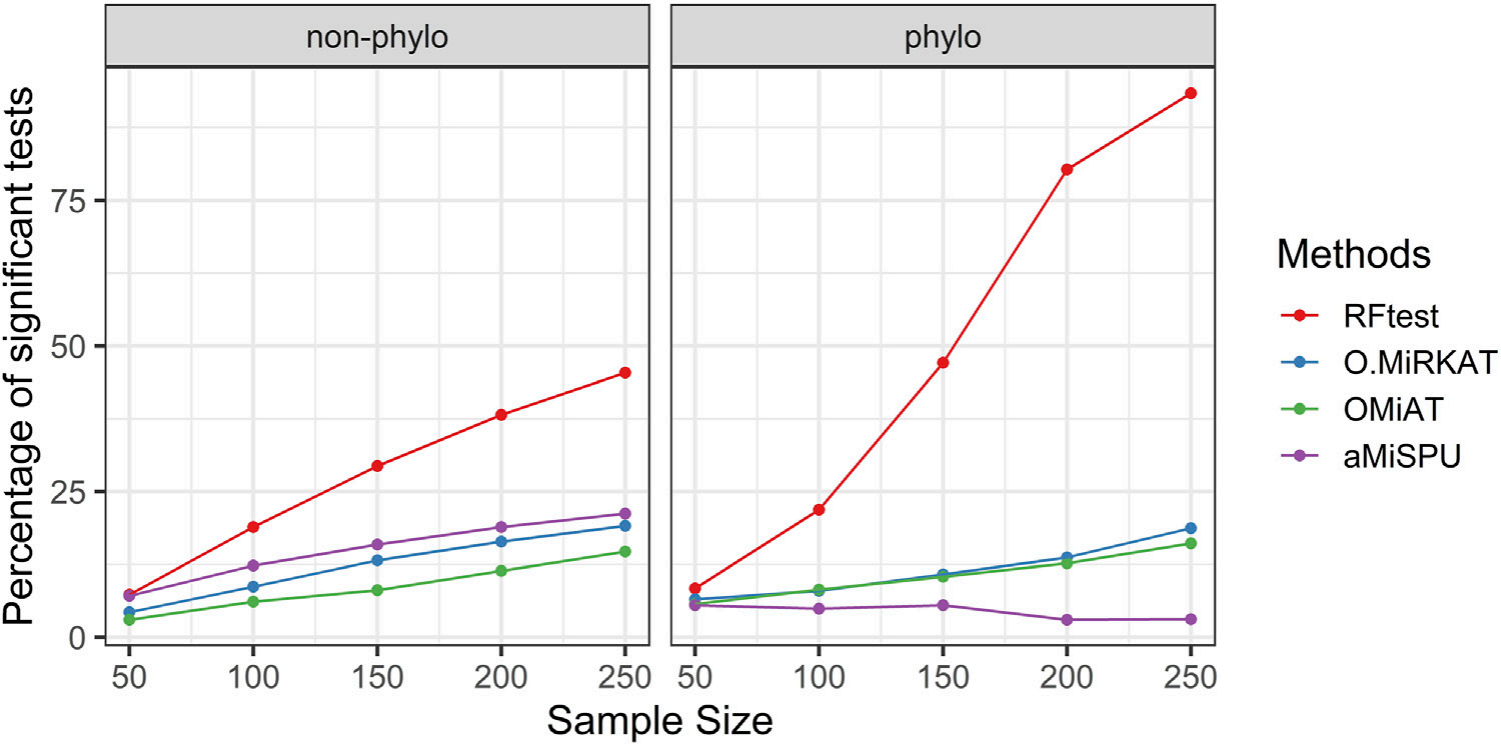
Power comparison among the competing methods when there was interaction between two microbial groups. The outcome variable was binary, and two signal types, phylogenetic and non-phylogenetic, were investigated. The two microbial groups comprised 13% and 15% of the total OTUs.

In scenario S4, where we simulated interaction effects between OTU clusters, we observed that while the RFtest was a leading method in this scenario, the pattern differed for a binary and continuous outcome. For a binary outcome, RFtest could effectively detect interactions between two phylogenetic clusters or non-phylogenetic groups at a relatively larger sample sizes (Figure 4). Meanwhile, the competing methods appeared powerless for both phylogenetic and non-phylogenetic signals (Figure 4). For a continuous outcome, RFtest could powerfully detect the association for both types of signals (Supplementary Figure S10). Meanwhile, the optimal MiRKAT and the OMiAT became considerably more powerful than the binary case under a non-phylogenetic signal (Supplementary Figure S10); however, they remained underpowered under a phylogenetic signal (Supplementary Figure S10).

**FIGURE 5:**
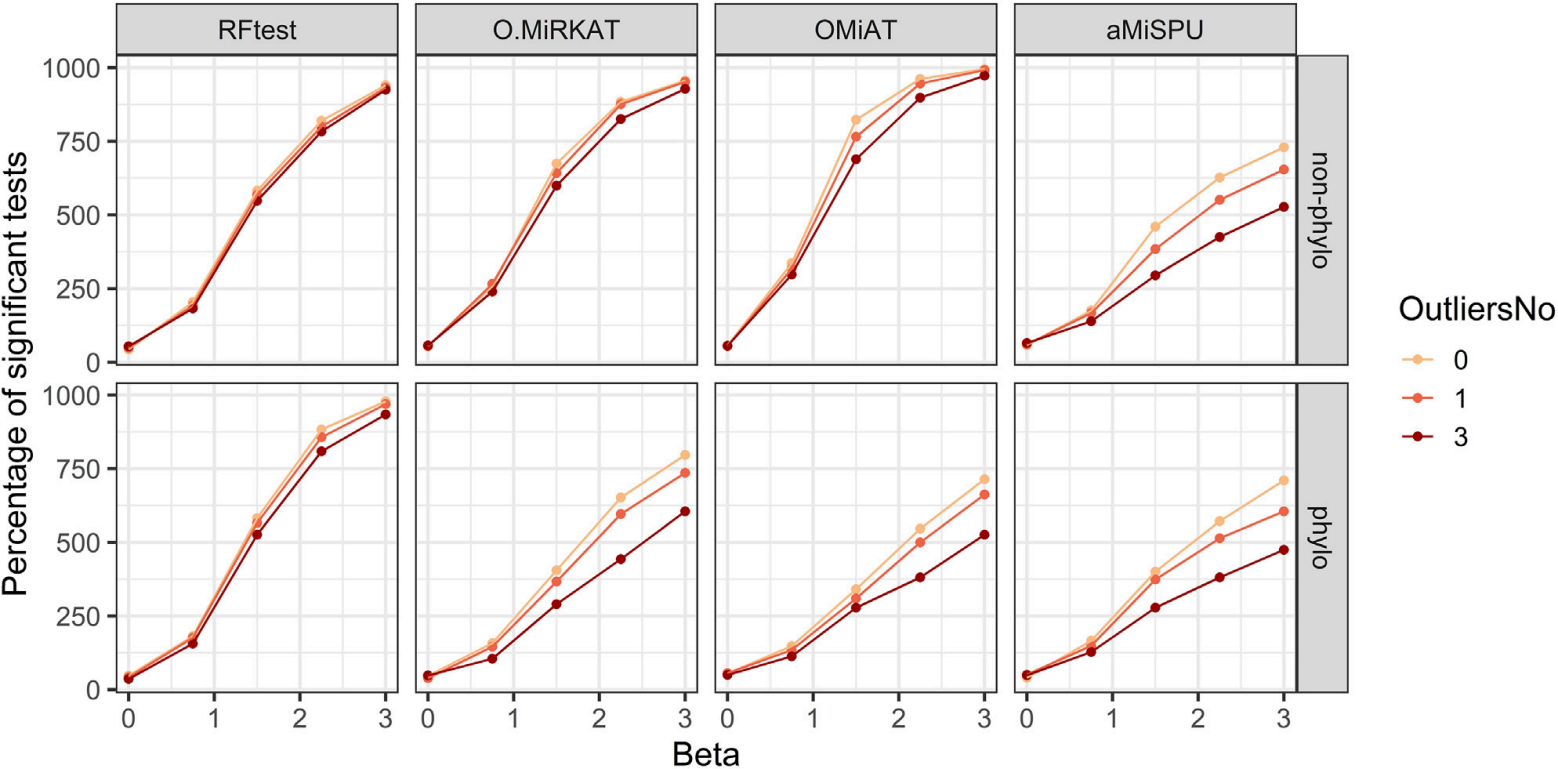
Power curves of the competing methods when outliers were present. The outcome variable was binary, and two signal types, phylogenetic and non-phylogenetic, with a density of 15% were assessed. Zero to three outlier samples were included.

In scenario S5, we simulated one and three outliers to assess the reduction in power when outlier samples were present. The results indicated that RFtest was the most robust among the competing methods, and that the presence of several outliers did not affect the power much for both binary and continuous outcomes with phylogenetic or non-phylogenetic signals, while the power of other methods might be considerably reduced (Figure 5; Supplementary Figure S11).

### 3.2 Real Data Analysis

In this section, we aimed to compare the results of RFtest, optimal MiRKAT, aMiSPU, and OMiAT in real-world examples. We re-analyzed the relationship between outcome variables and microbiome profiles in two published datasets. The first example was taken from a study on the throat microbiome (Charlson et al., 2010). That study investigated the effect of smoking on human microbiota in the upper respiratory tract. While detailed information of sample collecting and data processing procedures can be accessed from Charlson et al. (2010), a summary is provided here. Nylon-flocked swabs were taken from the nasopharynx and oropharynx of 62 healthy subjects, including 33 non-smokers and 29 smokers. From each swab, DNA was extracted using the QIAamp DNA Stool Minikit (Qiagen) and the V1–V2 region of the 16S rRNA was amplified. Thereafter, this 16S rRNA was sequenced using a 454 Life Sciences Genome Sequencer FLX instrument (Roche). The sequence reads were denoised (Quince et al., 2009), analyzed using the QIIME pipeline (Caporaso et al., 2010), and clustered into OTUs at 97% similarity using UCLUST (Edgar, 2010).

In the original study (Charlson et al., 2010), the association between smoking and the respiratory tract microbiome was tested by Permutational Multivariate Analysis of Variance (Anderson, 2001). based on weighted and unweighted UniFrac distances (Lozupone and Knight, 2005; Lozupone et al., 2007). A difference in microbial community structure was reported between smokers and non-smokers (*p* < 0.05). In the present study, we re-analyzed the microbiome data and found consistent results with previous studies (Charlson et al., 2010; Zhao et al., 2015; Wu et al., 2016). When no covariate was considered, the *p*-value estimated by the RFtest was 0.001 while those of the optimal MiRKAT, the OMiAT, and the aMiSPU were 0.006, 0.008, and 0.009, respectively. When biological sex was included as a confounder, the estimated *p*-values became 0.002, 0.009, 0.010, and 0.005 for the RFtest, the optimal MiRKAT, the OMiAT, and the aMiSPU, respectively. The RFtest provided more significant *p*-values in general, while all competing methods rejected the null hypotheses at a significance level of 0.01.

Another relevant example was taken from a study of the distance–decay relationship in microbial ecology (Xue et al., 2021). This relationship can be portrayed as relatedness of microbial communities decreases as their spatial distance increases (Hanson et al., 2012). In brief, surface soil was collected intact from a paddy field in Wenling, Zhejiang Province, China (28°21′ N, 121°15′ E) in November 2017. From the sample, a soil cube (2.0 cm × 2.0 cm × 2.0 cm) was selected and further divided into 4 × 4 × 4 cubes of which each had sides 0.5 cm in length. DNA samples were extracted from these sub-cubes, and the V4–V5 region of the 16S rDNA genes was amplified and subsequently sequenced using an Illumina HiSeq platform. After removal of adaptors and quality control, 16s rDNA sequences were aligned using USEARCH11 (https://www.drive5.com/usearch/) and OTUs were clustered at 97% identity using UPARSE (Edgar, 2013). Finally, the microbial communities were rarefied to 41,752 sequences per sample.

As one of the original findings (Xue et al., 2021), a decreased community similarity, measured by 1 − Bray–Curtis dissimilarity (Bray and Curtis, 1957) between microbial communities, was observed as the spatial distance increased in the 64 sub-cubes (Mantel test, *p* = 0.001). Herein, we re-examined this distance–decay association using the RFtest *via* an assessment of microbial changes along each spatial axis of the *xyz*-coordinate defined in the study of Xue et al. (2021). We found a similar result that the microbiome was associated with the *x-* and *y*-axes, and *p*-values by the RFtest were 0.001, 0.001, and 0.310 for the *x*-, *y*-, and *z*-axes, respectively. Those of the optimal MiRKAT were 0.011, 0.001, and 0.618, respectively; those of the OMiAT were 0.001, 0.001, and 0.265; and those of aMiSPU were 0.006, 0.001, and 0.135. While all methods discovered a statistically significant association between microbial changes and the *x*-axis, the RFtest reported a more significant *p*-value than the optimal MiRKAT and the aMiSPU, rejecting the null hypotheses at a significance level of 0.01.

## 4 Discussion

Random forest has been one of the most successful machine learning methods for microbiome data (Marcos-Zambrano et al., 2021). The superior predictive performance of random forest is due to its ability to model a complex nonlinear relationship between the microbiome and the outcome, to capture high-order interactions among taxa, and to accommodate a large number of taxa. In this study, we proposed a random forest-based test (RFtest) to assess the association between the microbiome and an outcome variable, borrowing the strengths of random forest in prediction. In RFtest, we incorporated phylogenetic structure by creating features that accumulate OTU abundance along the branches of the phylogenetic tree and used residual permutation to address covariates. Simulation results showed that RFtest could control type I error rate at the desired level with or without confounders (Table 1). This approach was closely linked to the naïve approach (Supplementary Figure S2); however, the naïve method could not address covariates, which limits its use in real-world applications.

Our benchmarking study further revealed that RFtest had a clear edge over the competing methods to detect phylogenetically clustered signals (Figure 1; Supplementary Figure S11). This is because our approach incorporates topological information of a phylogenetic tree *G* into random forest *via* creating features that accumulate leaf OTU abundances. This strategy could also be explored in other machine learning algorithms to capture a clustered signal. Conversely, when the signal OTUs are randomly distributed in the phylogenetic tree, the OMiAT (Koh et al., 2017) and optimal MiRKAT (Zhao et al., 2015) may become a better choice than the RFtest (Figure 1A; Supplementary Figure S7A). Though non-phylogenetic signal cases were less advantageous to RFtest, we consider that the superior power of RFtest for phylogenetically clustered signals may be practically more important, since phylogenetic signals are extensively observed in microbiome studies, and phylogenetic approaches are of particular interest in microbiome analysis (Washburne et al., 2018).

Our simulation results also demonstrated the robustness of RFtest to outliers and its adaptivity to various types of associations (Figures 3–5; Supplementary Figure S9–11). Microbiome composition is highly variable, which would largely be ascribed to stochasticity rather than explained (Clooney et al., 2021). Such large biological variation might consequently result in several outliers in a study. Remarkably, outliers affected the power of RFtest minimally, and RFtest was the most robust method to outliers in our benchmarking study (Figure 5; Supplementary Figure S11). Moreover, microbial communities have been portrayed as a complex ecosystem, in which its components closely interact with each other (Zengler and Zaramela, 2018). These interactions are generally categorized into two groups—beneficial and neutral relationships, such as mutualism and commensalism, and antagonistic relationships, such as competition and predation (Little et al., 2008). For mutualism and commensalism, they can be depicted as a non-linear, positive correlation between bacterial lineages and the outcome variable **Y**. For antagonistic relationships, a possible signal indicating competitive exclusion, denoted by **Y** = 0, would occur when one of two lineages overwhelms the other, denoted by **X**_1_ (+), **X**_2_ (–); otherwise, **Y** = 1 when **X**_1_, **X**_2_ (+) or **X**_1_, **X**_2_ (–). Therefore, they would be identified as interaction effects. Notably, our results showed that the RFtest was efficient in discovering a non-linear relationship (Figure 3 & S9) as well as an interaction effect (Figure 4 & S10). Given the relatively high performance of the RFtest under these complex conditions (Figures 3–5, **S9**, **S10** & **S11**), it may be projected that the RFtest can be flexibly applied to a wide range of data structures to ascertain associations between a microbiome profile **X** and an outcome variable **Y**.

There are several limitations for our proposed method. First, because of the use of bootstrapping in the random forest algorithm, RFtest can be computationally intensive. For example, it took 68 s and 70 MB in memory using a single core on a laptop computer to test the dataset of throat microbiome in our first real data example, compared to 2–4 s and 60–100 MB memory usage of its counterparts. Although computation is usually not a problem for a small dataset, more time would be required for larger datasets. The computation time of random forest increases linearly with the number of variables, i.e., *p*, and approximately linearly with the sample size *n* (Wright and Ziegler, 2017). To accelerate the computation of RFtest, we have implemented parallel computing in our software, where each permutation could be run in parallel. Moreover, we could perform sparsity-based filtering to reduce the number of input features to speed up the computation, without affecting the power much (Supplementary Figure S4). Another limitation may be that current random forest test could not as effectively identify random, non-phylogenetical signals as OMiAT (Figures 1A,B; Supplementary Figures S7A,B). Increasing the power for non-phylogenetic signal is our future direction of research, for example, by leveraging multiple weighting schemes in RFtest from external data with an omnibus test (Li et al., 2020).

## Data Availability

Publicly available datasets were analyzed in this study. The package “RFtest” was implemented on the R platform, which can be found on GitHub (https://github.com/Lujun995/Random-forest-test-RFtest). The source code of the simulations in the present study is available at GitHub (https://github.com/Lujun995/RFtest-Simulations).
