# Supplementary material for: RFtest: A Robust and Flexible Community-Level Test for Microbiome Data Powerfully Detects Phylogenetically Clustered Signals
Source: Front Genet. 2022 Jan 24;12:749573. doi: 10.3389/fgene.2021.749573 (PMC8819960; doi:10.3389/fgene.2021.749573)

**RFtest: A Robust and Flexible Community-level Test for Microbiome Data Powerfully Detects Phylogenetically Clustered Signals**

**Lujun Zhang**^1, 2^, **Yanshan Wang**^3^, **Jingwen Chen**^4,*^, **Jun Chen**^5,*^

^1^ Department of Biostatistics and Bioinformatics, Duke University School of Medicine, Durham, NC, USA

^2^ Institute of Soil and Water Resources and Environmental Science, College of Environmental and Resource Sciences, Zhejiang University, Hangzhou, China

^3^ Department of Health Information Management, University of Pittsburg, Pittsburg, PA, USA

^4^ Department of General Surgery, Zhongshan Hospital, Fudan University, Shanghai, China

^5^ Department of Quantitative Health Sciences, Mayo Clinic, Rochester, MN, USA

* Riceawen@163.com, [chen.jun2@mayo.edu](mailto:chen.jun2@mayo.edu)

**Fig. S1**. Power comparison among different methods to calculate the error rates of random forest. The outcome variable is binary, and phylogenetic and non-phylogenetic signals with a density of 15% were used. Abbreviations: Training, training error; OOB_P, out-of-bag error with probabilistic predictions; OOB_noP, out-of-bag error with binary predictions; 0.632, 0.632 error; 0.632+, 0.632+ error.


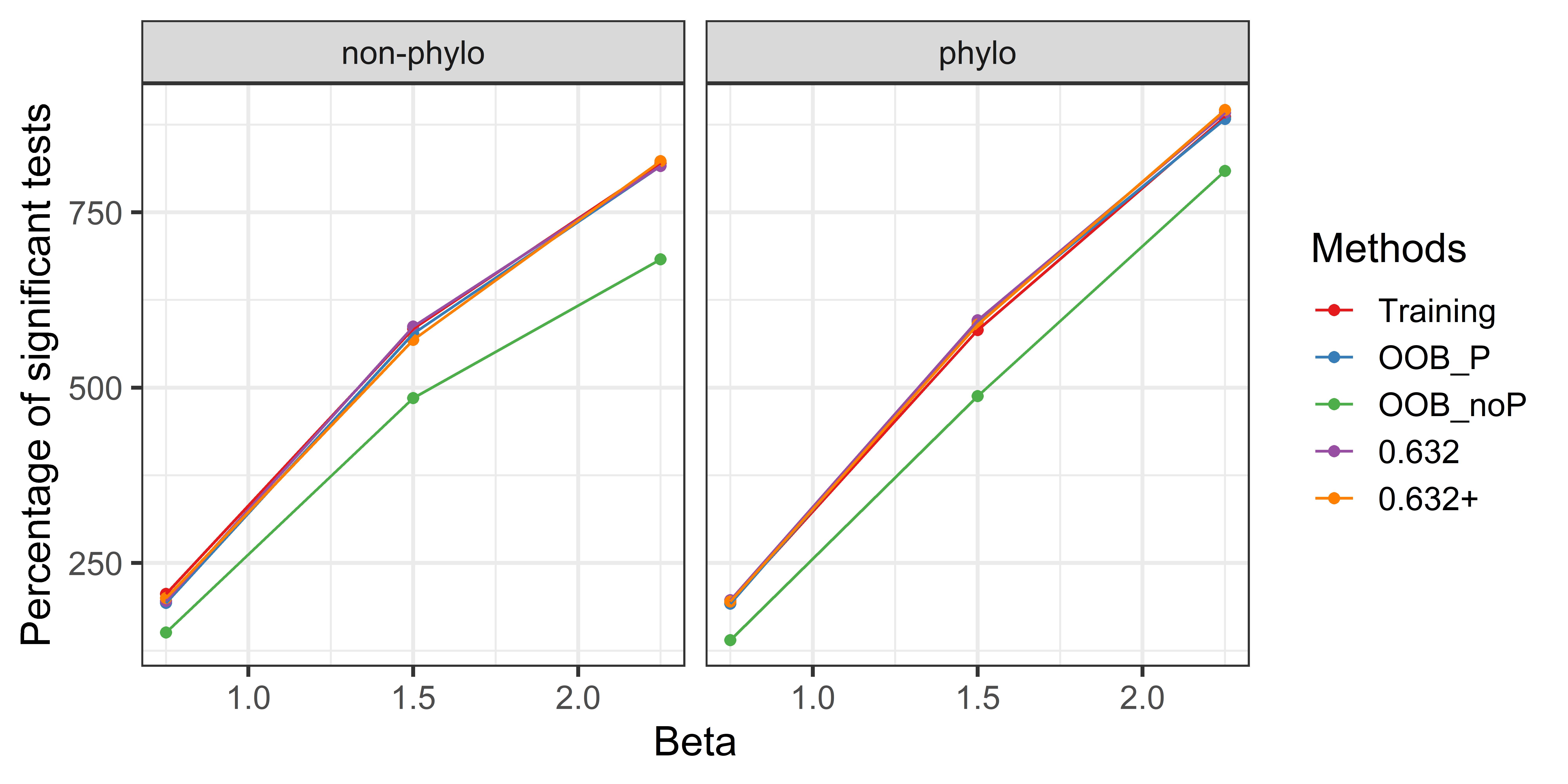


**Fig. S2**. Comparison between *P*-values generated by the naïve and the permutation approach.


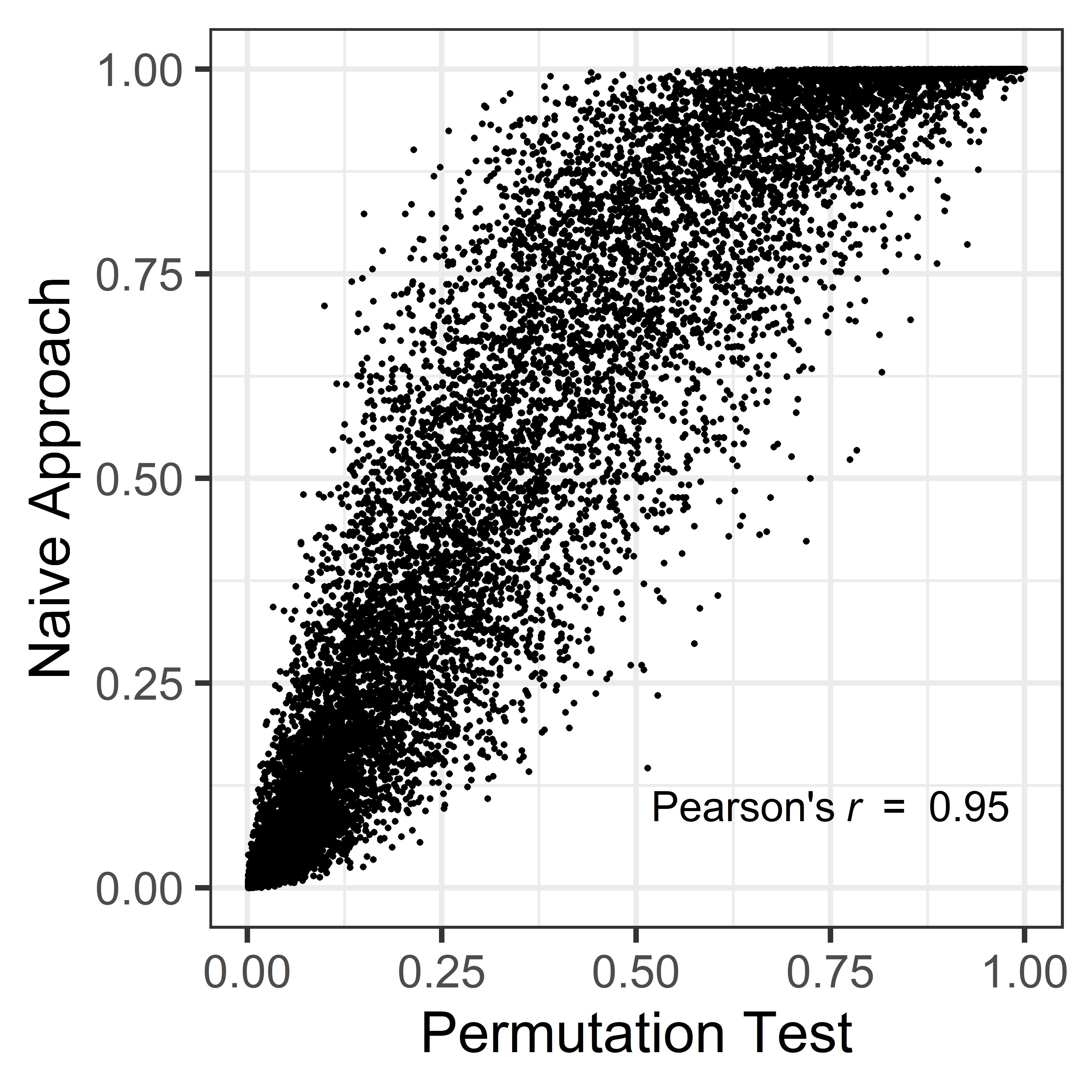


**Fig. S3**. Power comparison between the naïve and the permutation approach. Simulation settings were the same as in Fig. **S1**.


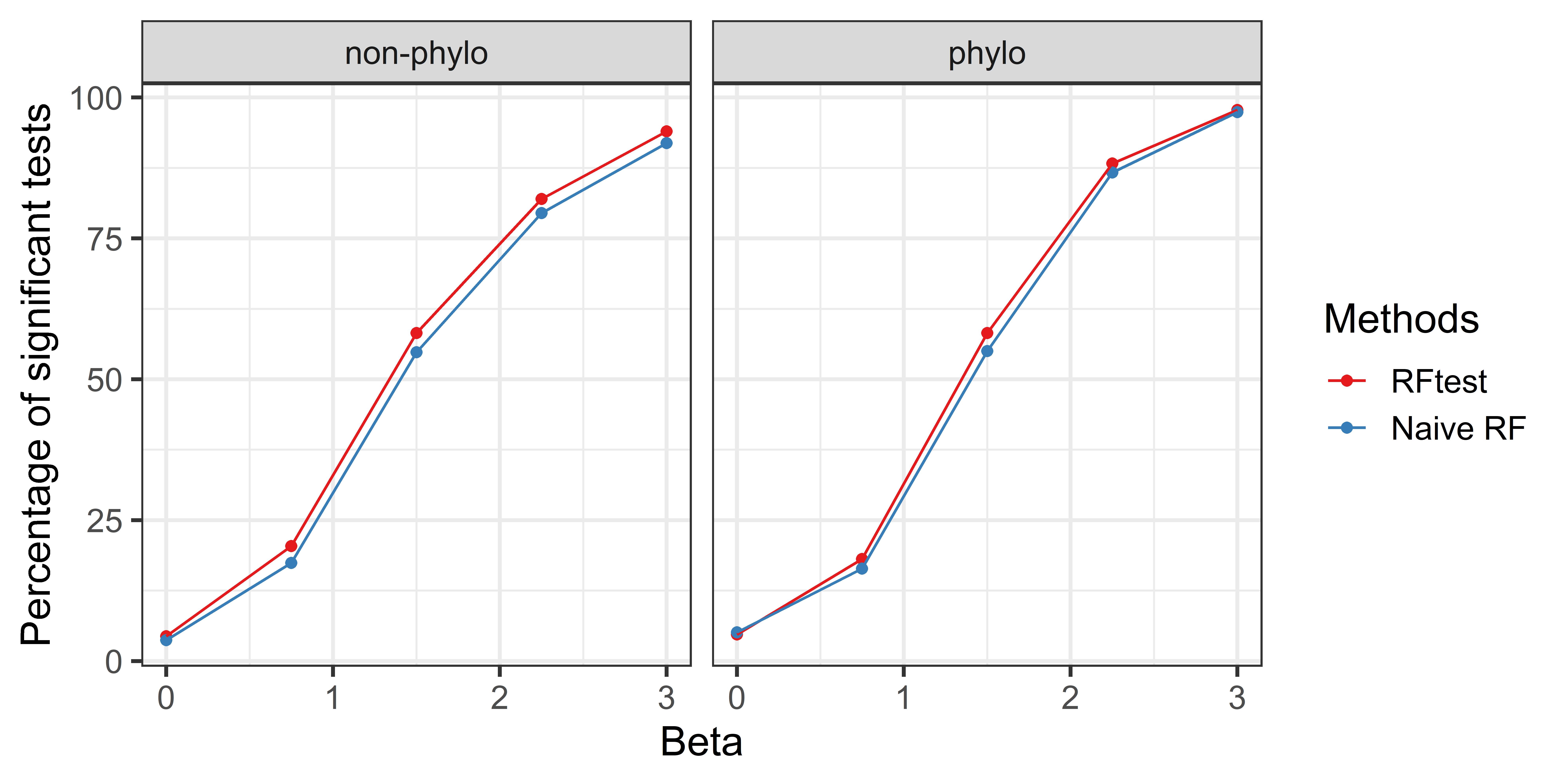


**Fig. S4**. Power comparison among different ways to engineer the features. The outcome variable is binary. Two signal types, phylogenetic or non-phylogenetic, with a density of 5% or 15% were investigated.


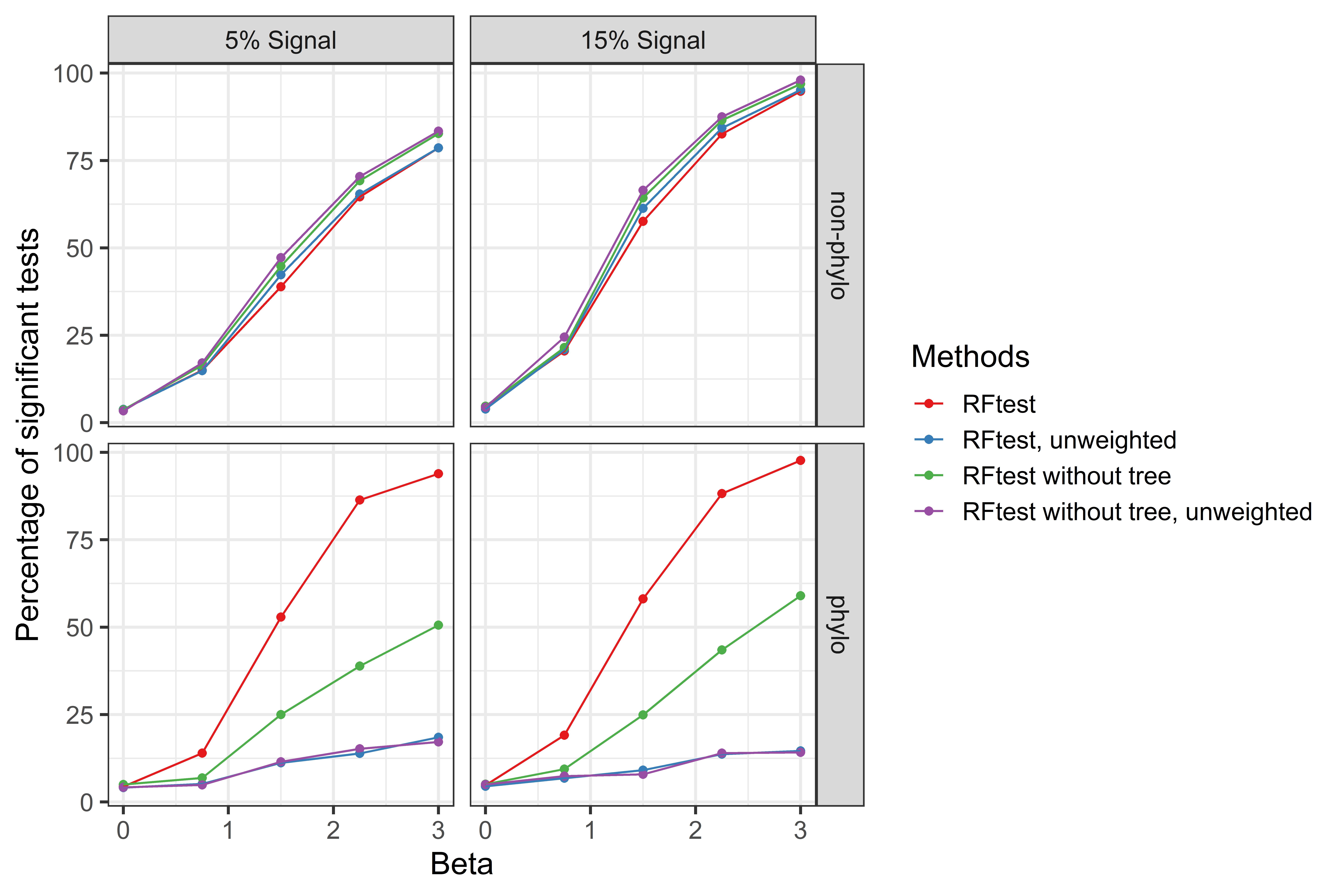


**Fig. S5**. The effect of sparsity filtering on the power. Simulation settings were the same as in Fig. **S1**. Abbreviations: RFtest.f4, OTUs present in less than 4% samples were excluded; RFtest.f10, excluded if less than 10%; RFtest.f20, excluded if less than 20%.


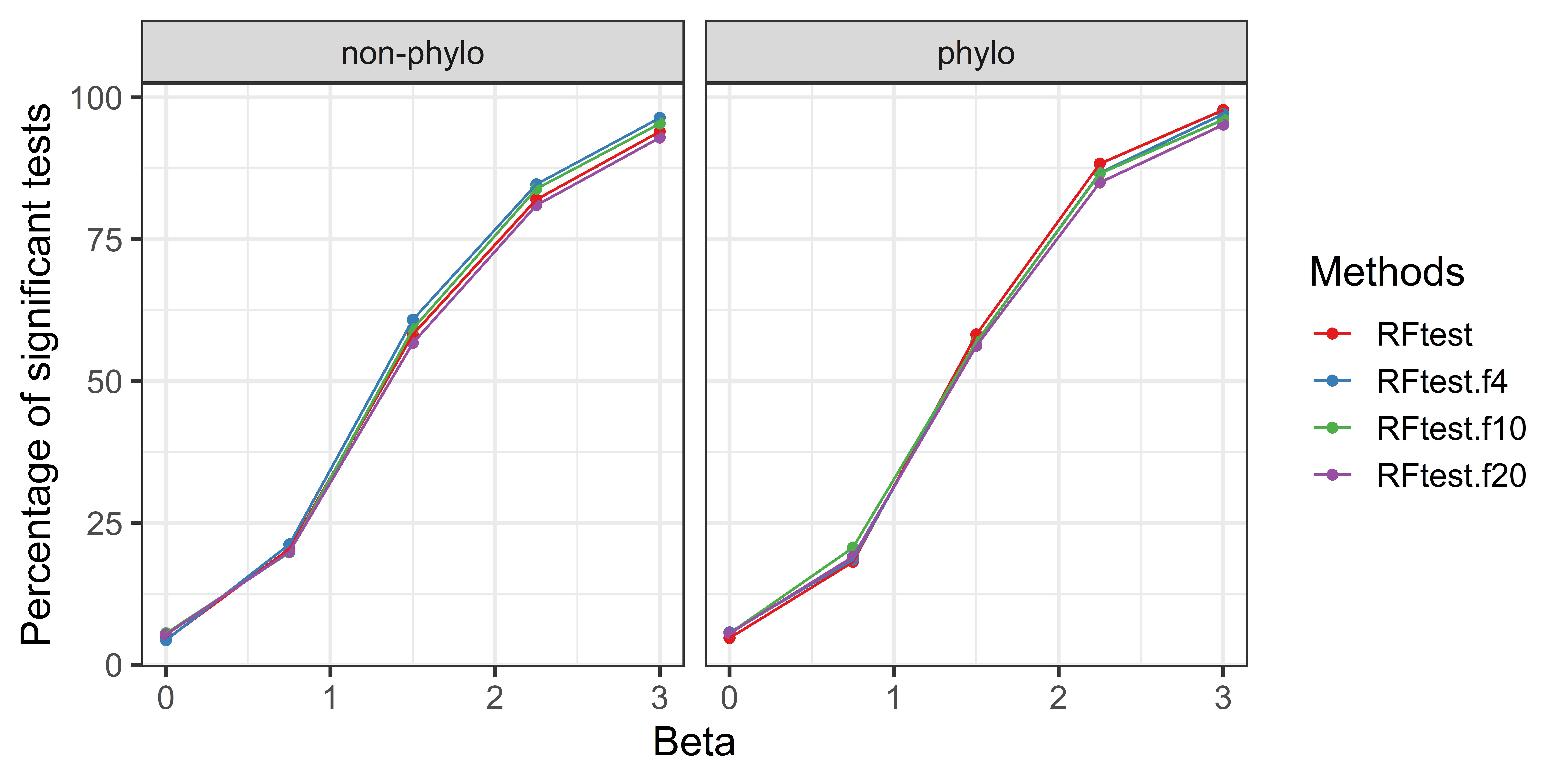


**Fig. S6**. The effect of the number of split variables used in random forest. Binary and continuous outcome variables were simulated, and phylogenetic and non-phylogenetic signals with a density of 15% were used.


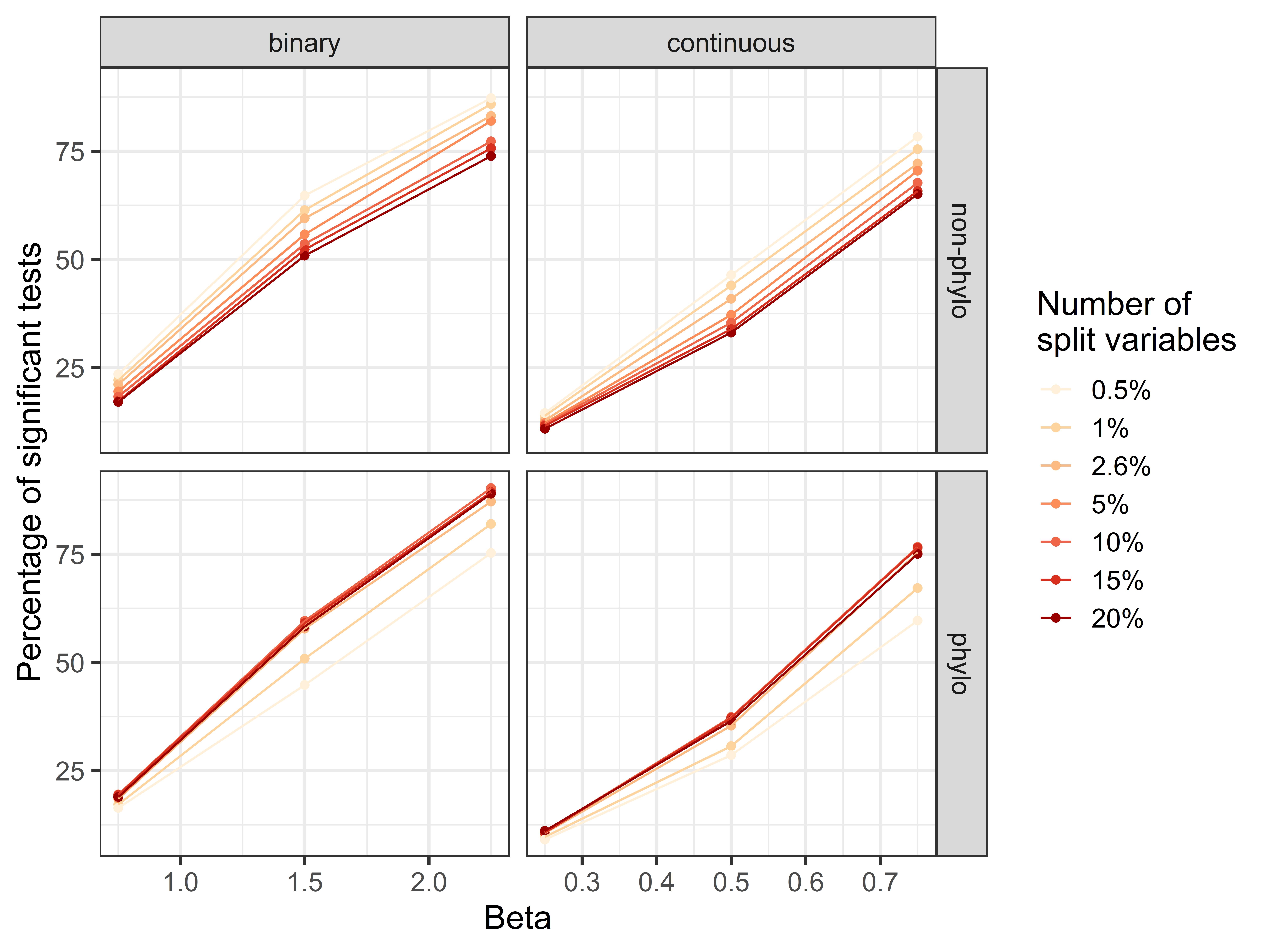


**Fig. S7**. The effect of the number of decision trees used in random forest. (a) An increased number of decision tress (presented in the form of its reciprocal) stabilizes the variance of the error rate estimate and slightly decreases the variance of the sampling distribution of the error rate. (b) Power comparison among random forest procedures using different numbers of decision tress. Two types of signals, non-phylogenetic and phylogenetic, with a density of 15% were used.


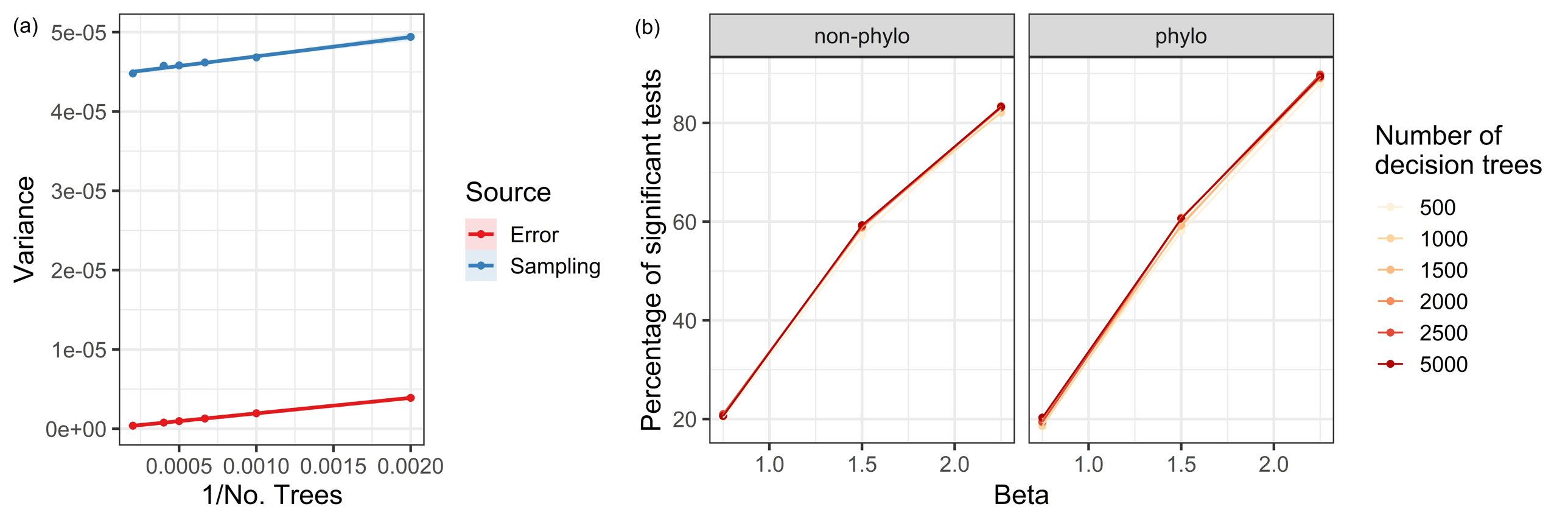


**Fig. S8**. Power comparison among the competing methods for a continuous outcome variable under different signal types and densities. Abbreviation: O.MiRKAT, optimal MiRKAT. (a & b) Random signals with a density of 5% and 15%, respectively. (c & d) Phylogenetically clustered signal with a density of 5% and 15%, respectively.


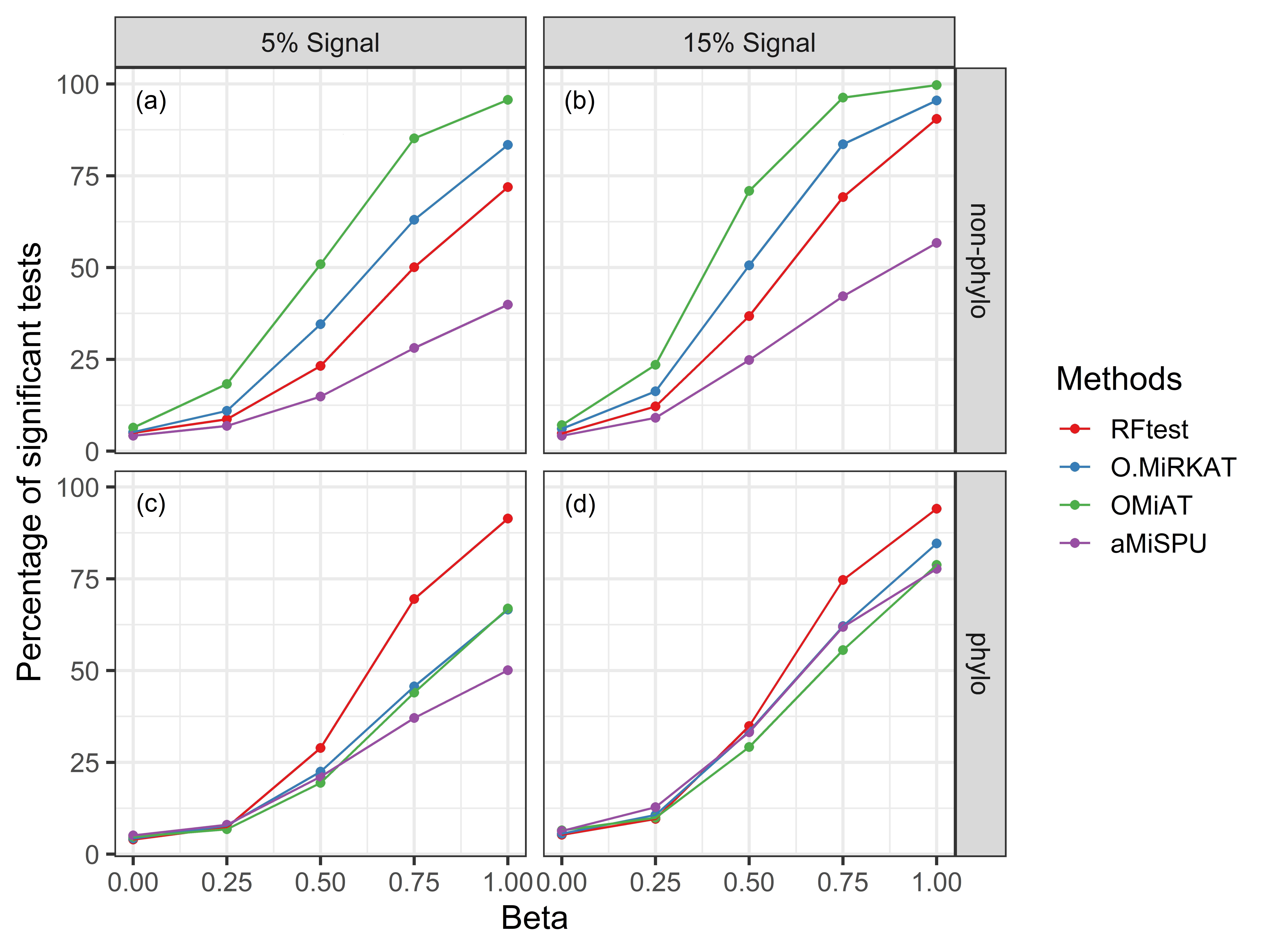


**Fig. S9**. Power comparison among the competing methods for a continuous outcome variable when signals are from seven major lineages. The lineage numbers correspond to node numbers in the phylogenetic tree used in simulation in the present study. These lineages spans ≥80% of the total OTUs and the total abundance.


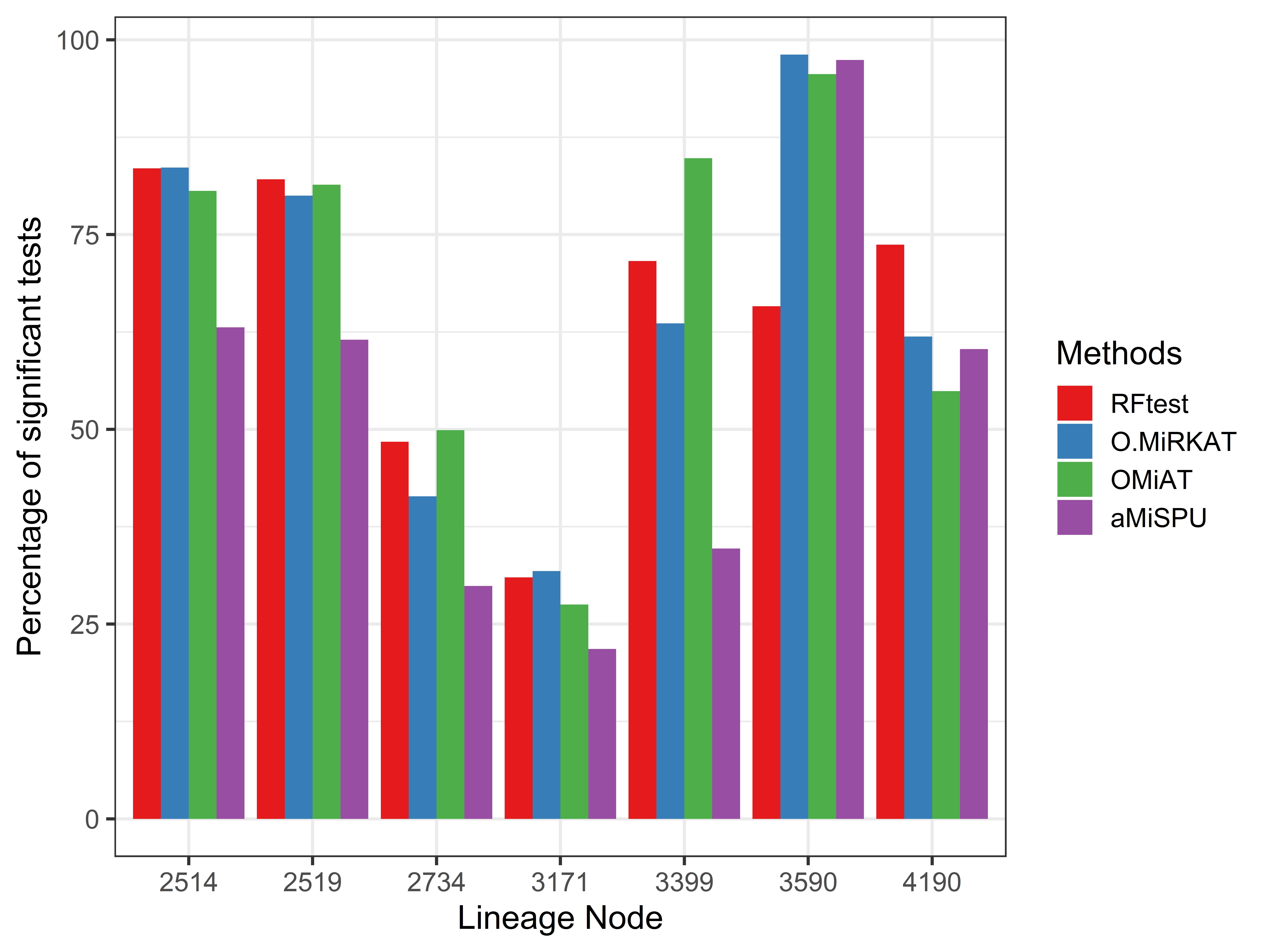


**Fig. S10**. Power comparison among the competing methods for a continuous outcome variable when **X** and **Y** are non-linearly correlated. The raw OTU abundance was log-linearly related to the outcome. Two signal types, phylogenetic and non-phylogenetic signals, with a density of 15% were used.


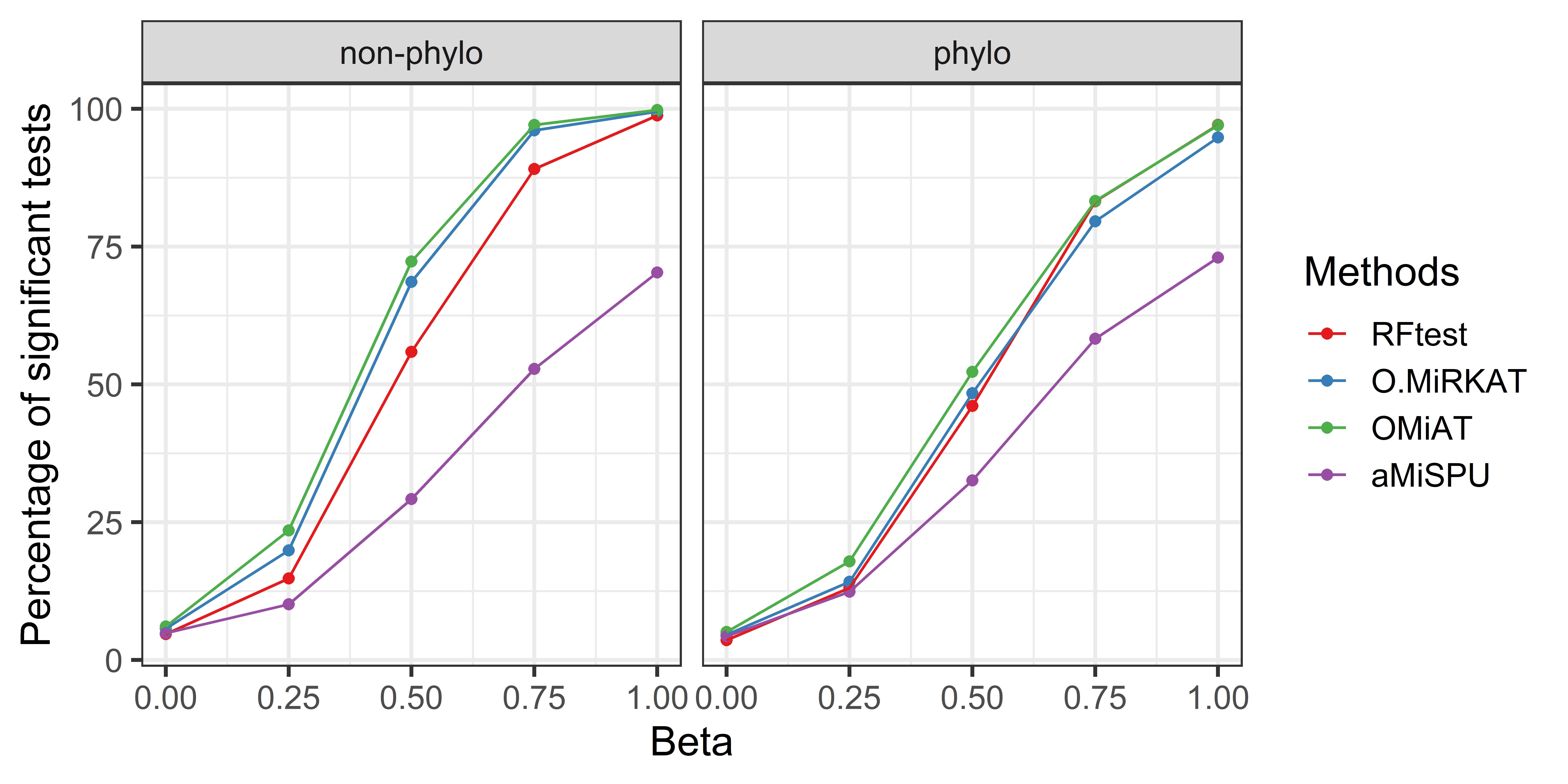


**Fig. S11**. Power comparison among the competing methods for a continuous outcome variable when there was interaction between two microbial groups. The outcome variable was continuous, and phylogenetic and non-phylogenetic signals were examined. The signals were generated by interaction of two microbial groups including 13% and 15% of the total OTUs.


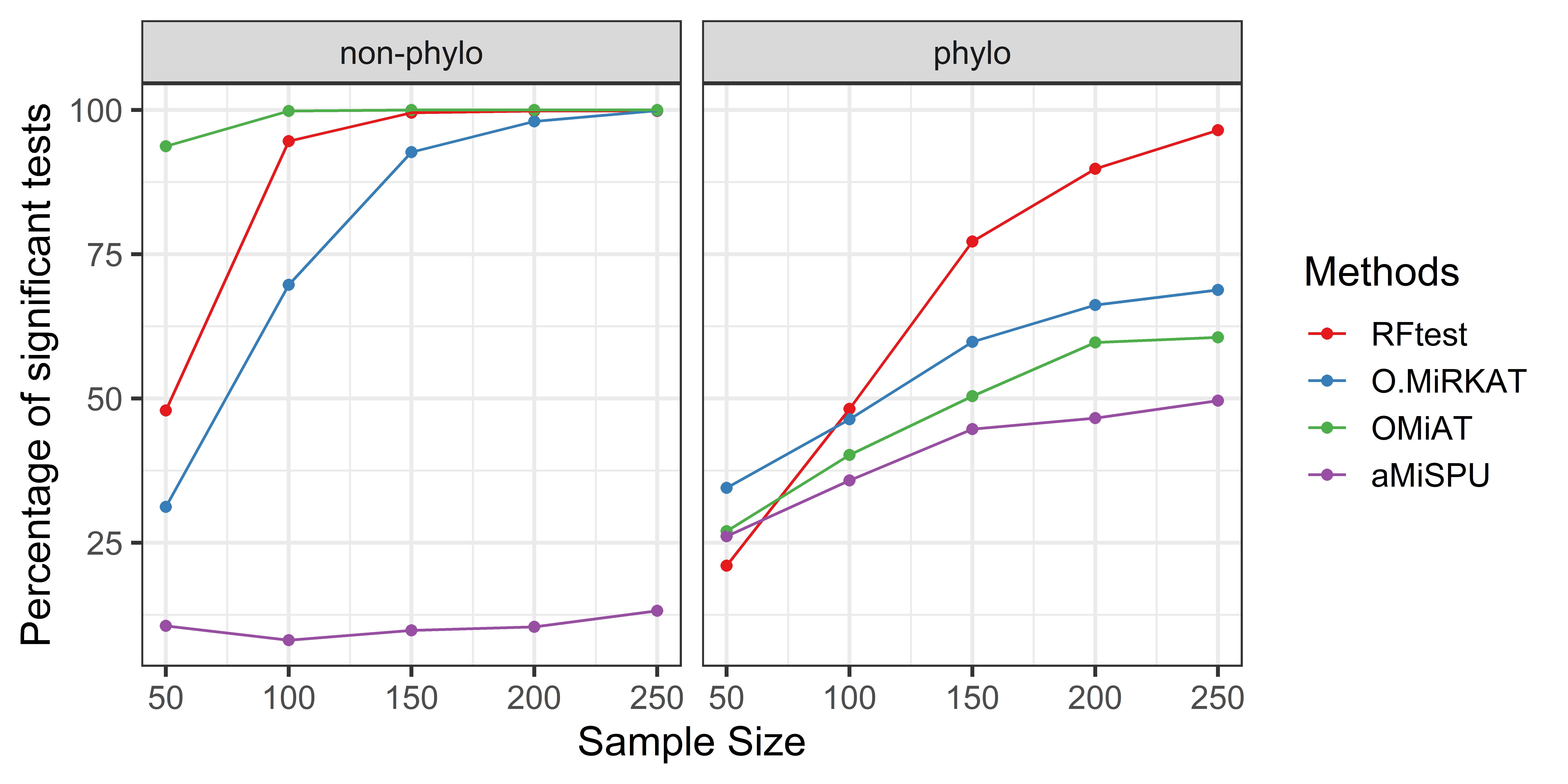


**Fig. S12**. Power comparison for a continuous outcome variable when outliers were included. Phylogenetic and non-phylogenetic signals with a density of 15% were used. Zero to three outlier samples were included.


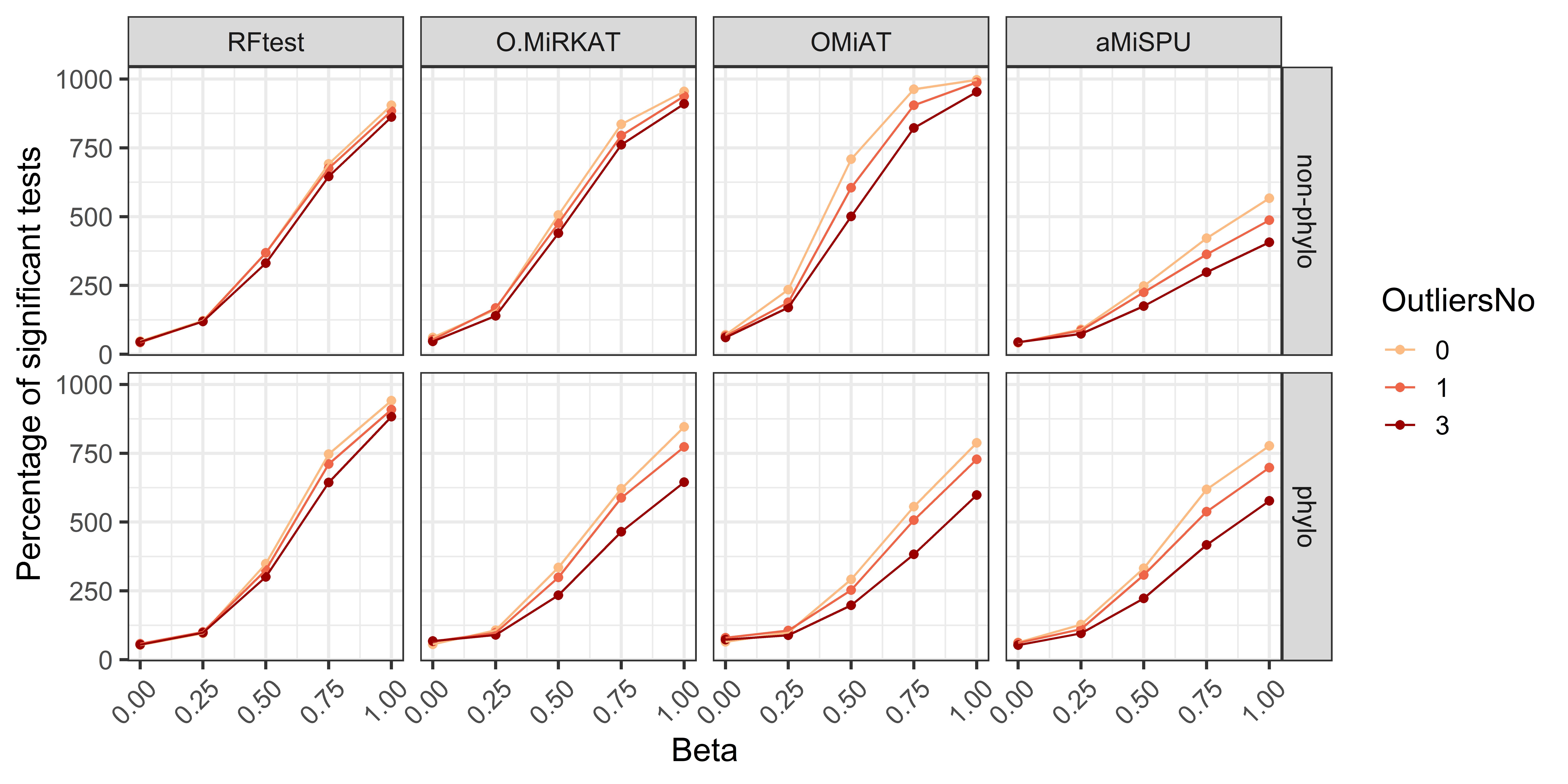

Supplement: Supplementary file 1 [file DataSheet1.docx]
